# An evaluation of family physicians' educational needs and experiences in health promotion and disease prevention in Poland and Lithuania - a qualitative study

**DOI:** 10.1186/1471-2296-12-13

**Published:** 2011-03-25

**Authors:** Tomasz Tomasik, Adam Windak, Alicja Domagala, Katarzyna Dubas, Linas Sumskas, Jerzy Rosinski

**Affiliations:** 1 Department of Family Medicine, Chair of the Department of Internal Medicine and Gerontology, Jagiellonian University Medical College, 4 Bochenska Street, Krakow, Poland; 2 Department of Health Policy and Management, Institute of Public Health, Jagiellonian University Medical College, 20 Grzegorzecka Street, Krakow, Poland; 3 Department of Health Economics and Social Security, Institute of Public Health, Jagiellonian University Medical College, 20 Grzegorzecka Street, Krakow, Poland; 4 Department of Preventive Medicine Medical Academy, Faculty of Public Health, Lithuanian University of Health Sciences, 9 Mickeviciaus Street, Kaunas, Lithuania; 5 Institute of Economics and Management, Jagiellonian University, 4 Lojasiewicza Street, Krakow, Poland

## Abstract

**Background:**

The aim of this study is to explore the views of family physicians/general practitioners about the most important competences in health promotion and diseases prevention and areas where these competences might be below the desired level.

**Methods:**

A qualitative, descriptive study, combining two data collection techniques, was conducted in two Eastern European countries in June and July 2009. Focus groups numbering 10 and 9 physicians, respectively, practising in various clinical settings, were held in Poland and Lithuania. Seven well-informed health care experts were recruited in both countries to provide information during the in-depth interviews. In both formats, questions were devoted to three main areas of health promotion and disease prevention competences: (1) educational, (2) clinical, (3) organisational. A qualitative content analysis was performed.

**Results:**

Lithuanian and Polish family physicians/general practitioners view preventive care as one of their main responsibilities. Among 3 areas of competences, participants identified clinical competences as the most important in everyday practice. They also acknowledged that organisational and educational competences might be below the level required for effective preventive care. Only clinical competences were indicated as sufficiently developed during under- and post-graduate medical education.

**Conclusions:**

In addressing current health promotion and disease prevention challenges, teachers of family medicine need to critically consider the training that currently exists for physicians. Development of a high-quality preventive service is not only a matter of proper education in the clinical field but also requires training in practice organisation and patient education.

## Background

Although the provision of preventive care belongs to the indisputable scope of the essential tasks of family physicians/general practitioners (FP/GPs) [1,2], very little is known about their self-perceived role in this field. The current paper attempts to provide new insights and fill in some of this gap.

There are considerable differences in the systems and organisation of primary health care (e.g. standard procedures, resources, forms of practice, number of available physicians in a given area, the role of providers, remuneration) in European countries.

In Eastern Europe, ongoing health sector reforms include implementation of family medicine, decentralization and privatisation [3]. There is a visible shift from care focused on treatment of acute and chronic diseases to care oriented towards prevention and the needs of patients, families and community. It is also expected that FP/GPs will gradually become more involved in health promotion and disease prevention (HP&DP) activities through effective screening, giving more frequent advice on lifestyle and prescribing pharmaceuticals when indicated [4]. Additionally, in some countries governments have introduced a law which obligates health care providers to undertake prevention-related tasks [5]. Until now, little has been known about the scope of, and obstacles to, preventive care in Eastern Europe, or about FP/GPs' views on these issues. Furthermore, health indicators show that there are important disparities between Eastern and Western European countries which may be directly related to preventive care [6].

In the years 2008-2010 a project focused on evaluation of HP&DP experiences in selected European countries was carried out by an international consortium. Within the framework of this project, supported by the Leonardo da Vinci programme [7], a qualitative study in Poland and Lithuania was conducted, with the aim of exploring the views of primary care physicians concerning: (1) the most important FP/GPs competences in HP&DP needed in their daily practice; (2) areas where competences acquired during vocational training may be below the desired level.

## Methods

### Design

A qualitative, descriptive study was conducted in Poland and Lithuania in which two data collection techniques were combined: focus groups and in-depth interviews.

### Setting

In Poland, qualitative data were gathered during the annual Congress of Family Medicine organised by the College of Family Physicians in Poland in Bialystok. A focus group was conducted on 6 June and interviews were carried out on 5 and 6 June 2009.

Kaunas University was the setting of the study in Lithuania. A focus group was conducted on 2 July and interviews in June 2009. Facilities belonging to the Faculty of Public Health were used for group and individual meetings.

### Participants

Only volunteers took part in the study. Participants were selected in a purposive manner. To facilitate recruitment in this study, administrative staff called potential participants. In Poland, the names were chosen from a list of the attendees of the Congress of Family Medicine; in Lithuania, from a list of family physicians cooperating with the Department of Family Medicine at Kaunas University. The selection process was aimed to include in the study physicians practising in various clinical settings, including private individual and group practices as well as public community health centres. When a FP/GP declined to participate in a focus group or in-depth interview, a doctor with similar characteristics was contacted.

A physician was considered eligible to take part in the focus group if he or she was currently practising as a FP/GP. Ten doctors were recruited in Poland and nine in Lithuania.

Seven well-informed health care experts recruited in both countries were engaged to provide information during in-depth interviews. Five of them were FP/GPs (one a chief of specialization, one a university teacher, two teachers in a practice, one responsible for supervision tasks in one region); one was an administrator representing the Ministry of Health; and one an administrator representing a medical university.

Oral informed consent was obtained from each participant.

### Data collection

#### Development of framework for questions about HP&DP

A framework for preparing questions about competences in HP&DP needed by FP/GPs in their daily practice was established as a part of the Leonardo da Vinci project [7]. As a first step, preliminary computer database searches supplemented with manual searches of print sources in Polish and Lithuanian were carried out. Then, at the beginning of 2009 a partners' meeting was organised and devoted to the scope of competences needed in daily practice. Two representatives from the partners' institutions (from Greece, Lithuania, Poland and the UK) participated in a panel discussion. Since the concept of competence has different meanings, for the purpose of this project it was defined as a combination of knowledge, skills and behaviour that enables FP/GPs to perform effective action in HP&DP within their practice. A brainstorm method was used to develop ideas. As a result all competences were divided into three main areas: (1) educational competences, (2) clinical competences, (3) organisational competences. For each of these areas several topics were generated. As the next step, all project partners conducted consultations in their countries in order to categorise and group the topics in three main areas of competences. Subsequently a consensus meeting was organised and for each main area three sub-areas were agreed upon. A developed framework is presented in table 1.

**Table 1:** Framework for questions about HP&DP.

Areas of competences Explanation	Main tasks of FP/GPs within the area	Sub-areas of competences
Area I: Educational Competences bound up with teaching the patient, his or her family and local community	Health promotion	1. Child and maternal health 2. Lifestyle 3. Environment

Area II: Clinical Competences bound up with providing preventive activities related to a particular disease	Disease prevention	1. Screening 2. Chronic disease management 3. Preventive interventions

Area III: Organisational Competences bound up with practice organisation	Provision of service	1. Information 2. Patient relationship 3. Local communities

#### Focus groups

In Poland and Lithuania a focus group of 10 and 9 physicians, respectively, was held and standard procedures were followed [8].

To ensure consistency between groups, a scenario consisting of 5 phases (opening, introductory, transitional, key, closing) was developed in Polish and afterwards translated into Lithuanian. The purpose of the study was described to the respondents during the introduction and basic rules for the focus groups were presented (e.g. no right or wrong answers, allow everyone to speak and value everyone's comments). Participants were informed that data would be handled confidentially. Emphasis was placed on the physicians' personal views and experiences and not on their theoretical knowledge.

The key questions included in the scenario were previewed in advance in a group of 4 FP/GPs in Poland to check if they could be readily understood. They are summarized in Table 2.

**Table 2:** Key questions in the focus groups and the in-depth interviews.

**The focus groups addressed the following questions**:	**The in-depth interviews addressed questions related to**:
1. General question: HP&DP competences indispensable in daily practice	1. The role of FP/GPs in HP&DP
2. Detailed questions about areas of specific competences (clinical, educational, organisational)	2. The most important areas in HP&DP for FP/GP competences
3. Additional questions in each of 9 sub-areas of competences if not discussed by the group	3. The most important (for FP/GPs):
	- clinical competences
	- educational competences
	- organisational competences
	4. The level of competences in specific areas

Experienced focus group facilitators specializing in health care led the group discussions using a pre-scripted scenario. Another individual observed the session, recorded the discussion on audiotape and took notes. Each group lasted about 90 minutes. Documentation of the focus group consisted of: (1) an audiotape, (2) a transcription, (3) notes of facilitators, (4) notes and comments of the observer.

#### In-depth interview

Interviews with 7 participants in Poland and 7 in Lithuania were conducted.

An interview protocol in Polish and Lithuanian (with rules to guide the implementation and administration of the interviews) was developed to increase the reliability of the findings. Questions are presented in table 2.

Since it was difficult to find experienced interviewers, individuals with higher education in health-related science were employed and instructed. The study coordinators in Poland and Lithuania prepared brief standardized training for the interviewers, including an introduction to the study and data collection techniques, an overview of the in-depth interviews, review of the data collection items, and practice in the use of the study protocol.

The interviews were conducted face-to-face, separately with each participant. At the beginning the purpose of the interview was explained and information about confidentiality was provided. Depending on the expressiveness of the interviewee and the flow of the interview, the interviewer asked more general or more detailed questions, but in every case aimed at covering the main topics.

After analyzing the costs (time, equipment, human resources) and potential benefits (reliability and validity of data) of the audio recording and transcription of the in-depth interviews it was decided that the interviewers would take only written notes. Interviews lasted 30-45 minutes. At the end of each interview, interviewers reviewed their notes. Afterwards they prepared a written report, which included (1) interview notes and (2) observations, comments or clarifications.

### Data analysis and interpretation

Data analysis, in accordance with qualitative content analysis [9], began separately in Poland and Lithuania following a common guide and using the framework presented in table 1. The study coordinators in each country ensured that all data (reports, notes, comments and recordings) had been obtained from the interviewers. Then the focus group transcription and the notes from the interviews were read and analysed by one investigator in each country. Relevant data segments were identified, coded and classified into nine sub-areas of competences presented in table 1. A report generated from each country analysis was prepared in English.

Next, an international team of investigators met and reviewed the reports. If ambiguity arose, information was checked from the original notes and an explanation or clarification was given by the national coordinator. The investigators checked the richness and variation of the data as well as number of times the specific competences were repeated. In such a way, they became "empirically" confident that saturation was achieved. They identified final issues for each of nine sub-areas. Additionally, they recognised a number of competences, which could be applied to different areas, rather than a single area.

This study involved no patients or human material, was lawfully available and in compliance with the Helsinki Declaration.

## Results

### Characteristics of respondents

Respondents were mostly men (70.5% in Poland and 50% in Lithuania) with an average age of 44.1 years. On average, FP/GPs had 10.8 years of professional experience as primary health care physicians. Most of the doctors practised in urban settings. Fourteen of them had previous experience with hospital work. Administrators had an average of 14.1 years of professional experience. Categories of respondents are presented in table 3. None of them had previously been involved in a qualitative study.

**Table 3:** Categories of study respondents.

Professional category	Poland		Lithuania	
	
	Focus group (N)	In-depth interview (N)	Focus group (N)	In-depth interview (N)
Family physicians	10	5	9	5
Individual practice	3	2	0	0
Group practice	6	2	6	3
Community health centres	1	1	3	2
Administrators	0	2	0	2
Government		1		1
University		1		1

Overall	10	7	9	7

### The most important competences in HP&DP

Both focus group and interview respondents expressed broad agreement that HP&DP is one of the most important competences and responsibilities of FP/GPs provided in everyday practice and one, which is also expected by patients. It was perceived as equally important as such activities undertaken in a practice as diagnosis and treatment. Words used by study participants to describe this perception were as follows:

*"...important...", "...fundamental...", "...crucial...", "...basic...", "... pillar...", "...key...", "...main..."*.

Among 3 areas of competences (educational, clinical, organisational), participants acknowledged clinical competences as the most important.

*" No one else can replace us (doctors) in delivering clinical work, whereas health education may be done by others... Others can organise our work too..." *(PL,M,44 = Polish doctor, male, 44 years old).

*"Patients come to me with complaints, so I think that taking medical histories and physical examination are important... and recognition of early signs of diseases*" (LT,M,52).

Within clinical competences none of the discussed sub-areas (screening, chronic disease management, preventive interventions) was indicated to be more important than the others. Moreover, there were more essential competences perceived in the clinical area than in the educational or organisational areas. These competences are listed in table 4.

**Table 4:** Most important FP/GPs competences in specific areas of HP&DP.

Areas of competences. Main tasks of FP/GPs within the area	Sub-areas of competences	Most important competences
Area I: Educational Task: Health promotion	1. Child and maternal health	(1) providing continuous care in antenatal, perinatal, early and late childhood; (2) facilitating referrals and consultation to higher level services for complex pregnancies; (3) family planning; (4) running monitoring programmes
	
	2. Lifestyle	(1) planning and implementation of individual and group educational activities alone and in collaboration with practice team members and other specialized services
	
	3. Environment	(1) identifying abnormalities in the family structure and functioning; (2) cooperating with different entities with a stake in health care in maintaining and protecting a healthy environment

Area II: Clinical Task: Disease prevention	1. Screening	(1) organising effective screening in practice; (2) linking screening to treatments available in the health care system
	
	2. Chronic disease management	(1) identifying early stages of chronic diseases (history taking and physical examination); (2) developing and implementing evidence-based strategies in order to prevent complications in chronic diseases; (3) coordinating services provided to patients by specialists and other health care providers; (4) managing co-morbidity, multimorbidity and patient complexity
	
	3. Preventive interventions	(1) identifying individuals at high risk for communicable and non-communicable diseases; (2) providing short-term interventions in addictions; (3) maintaining adequate immunization coverage; (4) counselling for modifying lifestyle/health behaviour; (5) providing referrals to specialists and other services

Area III: Organisational Task: Provision of services	1. Information	(1) gathering and retrieving medical information from practice using IT
	
	2. Patient relationship	(1) communicating with individuals; (2) developing and maintaining good relationships with patients and their families; (3) engaging patients in health promotion and disease prevention programmes
	
	3. Local communities	(1) applying community care measures on a local level to prevent diseases; (2) cooperating with professions involved in providing community care

During data analysis it became obvious that some physicians revealed a slight hesitation in distinguishing between cure of chronic diseases and tertiary prevention.

*"Does anybody know the borderline between a regular treatment of disease or long-term problem, diabetes, coronary or so on and tertiary prevention...?" (PL,F,34). "It is not easy to separate common clinical skills that I need to diagnose and treat patients from specific preventive skills" (PL,M,43)*.

In the area of educational competences there was high awareness among physicians of the importance of child and maternal health. Although some of them mentioned the role of other health professionals, e.g. obstetricians and midwives, they generally indicated this sub-area as more crucial than the other two. Respondents indicated that educational activities (individual and group) connected to lifestyle are also important. Among various environmental competences, only those related to functioning of the family and cooperation with different entities with a stake in health care were agreed to be important (see table 4).

Organisational competences were considered by doctors to be less fundamental than clinical. Nevertheless, they were described and discussed with attention to details. In this area, respondents believed that communication with patients and building good relationships with them and with their families are very important. It was uniformly expressed that new technology might be helpful in the collection and retrieval of medical information. Other important competences in the sub-area of local communities are listed in table 4. Typical opinions about organisational competences in HP/DP are cited below.

*"Communication is one of the most important GP competences, which is needed both for personal counselling and group sessions" (LT,M,45). "... the proper software can provide physicians with a tool to calculate a patient's health risk and to gather and retrieve information" (PL,M,37). "The most important organisational competence is to be able to collaborate with everyone who is useful in health promotion, I mean people involved in education, employment, environment, welfare, police, church. Hazardous factors are determined by broader social and community influences" (Pl,M,53)*.

During the interviews and focus groups some competences, which can be considered important in all areas and sub-areas of the HP&DP framework, were identified. These competences include the ability to: (1) develop professionally, (2) educate oneself in a continuous manner, (3) improve quality of care, (4) work in a team, (5) identify and solve problems, (6) set priorities in practice, and (7) provide holistic care.

Furthermore, apart from general or specific types of competences, the study participants recognized the most essential clinical problems and diseases that require preventive care. These conditions include: (1) cardiovascular diseases, (2) cancers, (3) infectious diseases, (4) pathological pregnancy, and (5) psychiatric disorders and addiction. Respondents believed that in relation to these problems each FP/GP should have a proper combination of knowledge, skills and attitudes that will enable them to provide preventive care in accordance with professional standards.

### Areas with inadequate competences

According to study participants, Polish and Lithuanian FP/GPs may have inadequate HP&DP competences in the areas of organisation and education. A prevalent belief was expressed that the existing system of under- and post-graduate education guarantees development of sufficient competences in clinical areas.

In the area of organisation a large gap may exist in competences related to teamwork.

*"There may be a lack of effective cooperation among the staff in practice" (LT,F,54). "More integrated teamwork, proper workload sharing with nurses and others is needed; efficient management, coordination, and mutual support rarely exist" (LT,F,35). "Teamwork is below a level that would assure quality and safety of care" (PL,M,44). "Doctors don't delegate tasks to others" (PL,F,44)*.

Also, competences necessary for proper cooperation with specialists from other disciplines or professionals from other sectors were perceived as possibly insufficient. Referring to this topic, study participants enumerated a large number of professionals FP/GPs should collaborate with.

*"The most important organisational competences which are lacking are an ability to collaborate with anyone who can be useful in HP&DP" (PL,M,44). "FP/GPs don't know how to coordinate with other local services" (LT,F,42). "Networking with appropriate services doesn't exist" (LT,M,45). "Ability to conduct a constructive dialogue with policy and decision makers, although valuable, is not undertaken" (PL,M,43)*.

Respondents expressed opinions that in the area of educational competences existing skills in changing patient behaviour might be insufficient.

*"Probably most of us (doctors) can give a patient information about lifestyle or something like that but the patient does what he wants" (PL,M,44). "I think that only modest changes in behaviour can be achieved in primary care and few patients follow advice; maybe multiple interventions are needed" (LT,F,54)*.

An additional issue to which participants of the study called attention was a possible lack of competence in physicians who, before implementation of family medicine in Poland and Lithuania, had practiced in primary care but had specialized in other medical disciplines, most often internal medicine or paediatrics. In the late 90s, these physicians completed specializations in family medicine after participation in short retraining programmes. In our study the opinion was often expressed that internists might provide inadequate preventive care for children, while paediatricians might do the same in caring for adult or elderly people.

## Discussion

### Main findings

This qualitative study showed that Lithuanian and Polish primary care physicians view HP&DP as one of their main responsibilities. From 3 areas of competences they identified clinical competences as the most important in everyday practice. Furthermore, these competences were indicated as sufficiently developed during under- and post-graduate medical education. On the other hand, doctors are aware that there are some areas of competences that may be below the level required for effective preventive care. These areas include patient education and practice organisation.

### Comparison with other studies

HP&DP competences vary significantly across individual countries in Europe, from well-established systems to countries with little development [10,11]. Since many countries do not have positions dedicated to health promotion, such responsibility is shared by all health providers, communities and governments [12]. The competences are also shaped by values, ethical norms and cultural or political factors unique to each specific country [13].

The position of FP/GPs within preventive care in relation to other professionals (nurses, health educators, public health specialists) continues to be debated. A number of associations, specialty boards, and even governments are taking actions to strengthen the competences of practising FP/GPs in preventive care [14]. As well, the findings of different studies support the involvement of practice nurses in cardiovascular risk management [15] and other preventive activities [16].

In our study HP&DP competences were divided into three areas: educational, clinical and organisational. Other authors used different categorizations, e.g. practical (requiring skills) and intellectual (requiring knowledge and understanding) competences or core, desired and specific (for setting or groups) competences [17,18].

The finding from our study that clinical competences are crucial in FP/GP practice corresponds to a publication by Arnold and Stern [19], who developed a framework for defining medical professionalism based on clinical competences. Also, Mueller has adopted a similar approach in his description of methods for teaching and assessing professionalism in the health care setting [20].

In our study, physicians from Poland and Lithuania identified two areas in which some shortcomings in competences may exist. The first area is health education, which relates strongly to health promotion and encompasses a wide range of actions at the individual, family and community levels. The importance of these competences was highlighted in The Galway Consensus Conference Statement [21] and their effectiveness in maintenance of health has been proved in many studies [22,23]. The second area of possible shortcomings is practice organisation, which includes such important issues as retrieving information, building relationships with patients and cooperating with the community. It is well known that the majority of currently practising physicians have never received formal training in practice organisation or quality improvement methodology that could guarantee sufficient competences in this area [24]. The lack of a systematic, organised approach within practices was shown by Leininger et al. as a reason why preventive services are not provided in primary care practices as often as they should be [25].

### Interpretation of findings

Results of this study illustrate the positive approach of FP/GPs to preventive care and the real-world complexity of HP&DP activities, and raise issues that are important for the successful education of doctors in preventive care in Eastern Europe. Since the study was conducted in Lithuania and Poland, the relevant historical background, specific context and culture of care should be taken into consideration. Until the mid-1990s, primary health care in both countries was provided by specialists (internists and paediatricians) who completed their vocational training in hospital wards and developed competences exclusively in the clinical area. Instead of teamwork, central planning and governmental orders were in place, while emphasis was put only on selected types of preventive intervention for example, vaccinations.

Family medicine was implemented 15 years ago, but specialists with limited organisational skills still provide an essential part of FP/GPs' vocational training. It is clear that, for them, accurate diagnosis and treatment is much more important than patient education.

In Poland and Lithuania difficulties with acquiring organisational and educational competences are related not only to vocational training in family medicine. Another example that highlights this problem clearly is a shortage of publications and CME courses in these areas.

In such a situation it is not surprising that FP/GPs who participated in this study described these two areas as inadequate.

### Strengths and limitations

Certain limitations should be taken into account when interpreting the results of this study. It should be emphasized that the presented study results come from a qualitative (not quantitative) study. Additionally, the sample of doctors participating in the study is rather small and not truly representative of the full range of physicians who routinely see patients in Poland and Lithuania. The doctors participated on a voluntary basis, had close relationships with a university or a professional organisation of FP/GPs and may be exceptionally motivated to provide high-quality care. Women were underrepresented in our study.

The number of conducted interviews was in accordance with a rough calculation of Brod M et al. who stated that when combining both focus group and individual interviews it is generally enough to conduct 3-4 focus groups in combination with 4-6 individual interviews to reach saturation [26]. Other authors who systematically documented the degree of the data saturation and variability found that saturation occurred within the first twelve interviews [27].

One could state that a lack of an audio recording of the in-depth interviews may be a limitation of this study. The verbatim transcriptions are clearly beneficial because they bring researchers closer to their data. On the other hand, within the current literature the use of field notes taken during and immediately after the interviews have also been reported as valuable [28,29].

It might be stated that the results of this study do not provide a definite conclusion. Nevertheless, they constitute a well-grounded basis for conducting further research. By combining two qualitative techniques, we were able to identify not only individual interpretative perspectives (in the in-depth interview) but also the views of groups and a variety of experiences or opinions (in the focus groups) [30].

### Implications of this study

We believe that our study provides some insights that will be important for practice, education and research.

Firstly, disease prevention strategy developed on a regional or local level should focus not only on raising community awareness or funding support but also on provider education, especially in non-clinical areas. When preventive programs are implemented on a health care level at which the organisational competences of doctors or nurses are limited, then clear guidelines or other supported measures should be prepared.

Secondly, teachers responsible for planning and providing education at different level should not confine themselves to clinical areas of HP&DP but ought to expand their efforts to include other important topics. Under-graduate teaching on preventive care still should be focused on clinical issues, whilst vocational training of FD/GPs should be extended in the areas of patient education and practice organisation. Moreover, individual FP/GPs who are involved in CME should also make an effort to fill the gap in HP&DP competences.

It is important to emphasize that this study offers no evidence that improvement of FP/GP competences in a specific area of HP&DP will improve health outcomes. Further quantitative research evaluating this topic is certainly needed in primary care in Poland, Lithuania and in many other Eastern European countries. Nonetheless, it seems important to understand how FP/GPs view their competences in preventive care.

## Conclusions

Taking into consideration the limitations of this study, we can conclude that FP/GPs from Eastern Europe view HP&DP as their main responsibility, although they are aware of areas in which their competences are limited.

There is a need to critically consider existing physicians' under- and post-graduate training in addressing current HP&DP challenges. Development of an extensive and high-quality preventive service in primary health care is not only a matter of proper education in the clinical field but also requires training in practice organisation and patient education.

Teachers of family medicine should be aware of possible shortcomings in HP&DP education and act to eliminate them.

## Competing interests

The authors declare that they have no competing interests.

## Authors' contributions

TT, AW, AD and KD were responsible for the conception and design of the study; KD, LS and JR collected the data and carried out the initial analysis; TT, AW, AD, KD and LS were responsible for final analysis and interpretation of data; TT and AW coordinated the study and wrote the first draft and subsequent revisions of the manuscript; all authors revised the manuscript critically, and read and gave final approval of the version to be published.

## Pre-publication history

The pre-publication history for this paper can be accessed here:

http://www.biomedcentral.com/1471-2296/12/13/prepub
